# Spatial localization of cathepsins: Implications in immune activation and resolution during infections

**DOI:** 10.3389/fimmu.2022.955407

**Published:** 2022-08-03

**Authors:** Elsa Anes, David Pires, Manoj Mandal, José Miguel Azevedo-Pereira

**Affiliations:** Host-Pathogen Interactions Unit, Research Institute for Medicines, iMed-ULisboa, Faculty of Pharmacy, Universidade de Lisboa, Lisboa, Portugal

**Keywords:** cathepsins, infection, inflammation, innate immunity, adaptive immunity, immune resolution

## Abstract

Cathepsins were first described, as endolysosomal proteolytic enzymes in reference to the organelles where they degrade the bulk of endogenous and exogenous substrates in a slightly acidic environment. These substrates include pathogens internalized *via* endocytosis and/or marked for destruction by autophagy. However, the role of cathepsins during infection far exceeds that of direct digestion of the pathogen. Cathepsins have been extensively investigated in the context of tumour associated immune cells and chronic inflammation. Several cathepsin-dependent immune responses develop in the endocytic pathway while others take place in the cytosol, the nucleus, or in the extracellular space. In this review we highlight the spatial localization of cathepsins and their implications in immune activation and resolution pathways during infection.

## Introduction

The term cathepsin (CTS) was initially used to refer to eleven human lysosomal proteases namely CTSs B, C (J), F, H, K, L, O, S, V (L2), X (P,Y,Z), and W (lymphopain) [reviewed in (1–3)]. They all belong to the group of cysteine cathepsins named after the presence of a cysteine amino acid residue on their catalytic site responsible for hydrolysis of peptide bonds (3). In addition to cysteine cathepsins, aspartic cathepsins D and E and serine cathepsins A and G, were also introduced into the lysosomal CTS family (4, 5).

CTS are synthesized as procathepsins and are targeted to the lumen of the endoplasmic reticulum (ER) *via* a signal peptide. They are later modified in the Golgi, being tagged for lysosome sorting, usually *via* mannose-6-phosphate receptors (MPR) (6–8). The tagged procathepsins are either directly or indirectly sorted to endosomes/lysosomes after escaping MPR and being secreted out of the cell (9–11). Around 5% of all CTSs are secreted out of the cell by the regular biosynthetic/secretory pathway (12).

Innate immune cells, such as macrophages, are able to rescue some of these extracellular CTSs to the lysosomes by expressing the cation-independent mannose 6-phosphate scavenger receptor (CI-MPR) (7, 8). There, in the low pH of the late endocytic vesicles they are processed and activated to the mature form (13).

Indeed, immune cells such as macrophages, neutrophils, natural killer cells or cytotoxic CD8^+^ T-lymphocytes, can store CTSs either in endocytic lytic granules or in secretory lysosomes, where exocytosis leads to delivery of CTS or their processed products to the extracellular environment (14, 15).

Lysosomal enzymes were also found in less common locations, such as the cytosol and the nucleus (16–20). The cytosolic release of mature CTS is observed as a consequence of controlled lysosomal membrane permeabilization (LMP) or as a result of a more drastic damage (15, 21). Regarding the trafficking of CTS to the nucleus, this is mostly a diversion from the biosynthetic pathway, through mechanisms involving alternative translation initiation of the nascent protein lacking a signal peptide targeting the ER (22). Another described mechanism is exon skipping that generates truncated CTS with modified signal sequences, enabling the retention in the cytosol (23) or their nuclear targeting (24).

**Figure 1:**
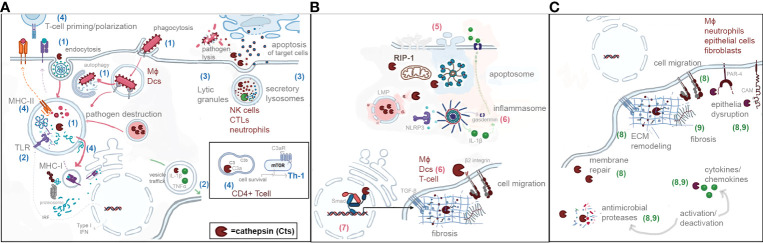
Schematic representation of the spatial localization of cathepsins and their roles in endocytic pathway **(A)** (blue numbers from 1 to 4), **(B)** cytosol and nucleus (pink numbers from 5 to 7), and **(C)** extracellular environment (green numbers 8 and 9). (1) Phagocytosis/endocytosis/autophagy. (2) Pattern recognition/cytokine activation. (3) Activation of proteases. (4) T-cell priming and polarization. (5) Programmed cell dead. (6) Inflammation. (7) Regulation of transcription. (8) ECM remodeling. (9) ECM inflammation during infection.

In this mini review, we will present recent advances in the understanding of the spatial localization of CTS and their implications during immune responses to infections. A general schematic representation is depicted in **Figure 1**.

## Cathepsins in the endocytic pathway

### Phagocytosis and autophagy

CTS perform major roles in phagocytosis/endocytosis and autophagy which are important cell autonomous immune mechanisms common to all cells (25). These innate mechanisms are prominent in professional phagocytes such as macrophages, and neutrophils, which constitute the first line of defense against pathogens. CTS mediate the destruction of theses pathogens due to their proteolytic activity at low pH, within the reducing environment of endolysosomes (26). It is not surprising that intracellular pathogens evolved virulence determinants to subvert the microbicidal mechanisms mediated by endolysosomal CTS as is the case for *Mycobacterium tuberculosis* (27–29), as well as for Salmonella, Brucella, Legionella or Chlamydia (30–33) or *Francisella novicida* (34). Autophagy intercepts the endolysosomal pathway (34–36) and may drive free cytosolic pathogens for destruction in lysosomes (37) or pathogens contained in vesicles (25, 38); both processes involving their entrapment in septin cages (39).

Indirectly, CTS regulate autophagy and may compromise intracellular pathogen clearance with direct implications on inflammation resolution and cell homeostasis (40–42). CTS S is required for autophagolysosome fusion events and its depletion results in accumulation of defective autophagosomes (43). CTS B suppresses the activity of a transcription factor required for expression of autophagy-related proteins (Atgs) by digestion of a calcium channel in the lysosomes (34). Also, the stimulation of the autophagy protein microtubule associated protein 1A/1B light chain 3 (LC3) is compromised by CTS K downregulation of endosomal TLR9 (44).

### Pattern recognition receptors and cytokine activation

Innate immune receptors, such as Toll-like receptors (TLRs), detect pathogen-associated or cell damage associated signatures (PAMPS or DAMPS) leading to secretion of inflammatory cytokines such as IL-1β and TNFα. CTS interfere with both mechanisms. CTSs B, L, F or S, by cleaving and processing the ectodomains of endosomal TLRs such as 3 and 9, allow recognition of nucleic acids from endocytosed pathogens (44–48).The ectodomain cleavage represents a strategy to restrict receptor activation to endolysosomal compartments and prevent TLRs from responding to self nucleic acids (48).

CTS activity was demonstrated to either directly activate or inhibit inflammatory cytokines. While spatial localization in endosomes was not clarified, CTS B has been shown to be required for posttranslational processing and trafficking of TNFα (49) containing vesicles, and their secretion in response to TLRs 2, 4 and 9 stimulation (50). IL-1β is a potent inflammatory cytokine that needs to be tightly controlled (51, 52). Several CTS are involved in IL-1β processing in the cytosol. However, in monocytes, which are professional IL-1β producers, caspase-1 and pro- IL-1β coexists with CTS within special secretory endolysosomes (53, 54). This colocalization in vesicles located in the periphery of the cell suggests a less acidic and degradative environment (55) and seems to provide a regulatory mechanism of CTS over the amount of caspase-1 and IL-1β that are secreted by monocytes. In conventional endolysosomes, IL-1β and their precursors are normally degraded. Thus, the lysosomal pathway mediates IL-1β secretion but also provides a shutdown mechanism when IL-1β secretion is no longer needed (53, 54). Moreover, the autophagic removal of IL-1β cell activators, such as intracellular DAMPs, NLRP3 inflammasome components, and cytokines, in lysosomes contributes for deactivating the inflammatory responses (56). Cathepsins as degrading proteases in lysosomes are major players in this inflammation resolution.

### Activation of other proteases

During innate immune responses, neutrophils are cells involved in extracellular and intracellular pathogen clearance. Their effector functions depend on the activation of azurophil granules, serine proteases such as CTS G, granzymes, and elastase, all synthesised as inactive zymogens and activated by CTS C (57). Regulation of these neutrophil serine proteases activation is tightly controlled by sustained inhibition of CTS C through its natural inhibitor, cystatins (57).

In natural killer cells (NK cells) or in cytotoxic T lymphocytes (CTL), CTS C is responsible for the activation of progranzymes, generating granzymes A and B in secretory lysosomes (58). After immune activation they are processed and delivered out of the cell, where they induce apoptotic death of infected cells (58). CTS B is particularly relevant for protecting CTLs from their cytotoxic cargo (59). In addition, granzyme B is involved in regulating the function and maintenance of T helper cell populations (60). In regulatory T lymphocytes, activated granzymes can eliminate autologous effector cells by apoptosis, indicating an important role accomplished by Treg cells in exerting their anti-proliferative effects leading to immune resolution (61).

### lymphocytes priming and polarization

During adaptive immune responses, T lymphocyte priming by antigen-presenting cells (APCs) requires the recognition of processed antigenic peptides bound to major histocompatibility complex (MHC). CTS are crucial for the generation of these antigenic peptides from exogenous antigens in the endocytic pathway, and thus for CD4^+^ T lymphocytes priming. Furthermore, MHC class II requires CTS mediated proteolysis for degradation of the invariant chain (Ii) that blocks MHC class II molecule peptide binding site (62, 63). CTSs S, F, and L are cysteine proteases particularly implicated in these processes, with Cts S and F major players in in APCs and the last in thymocytes (29, 42, 64–67). CTS have also been shown to generate antigenic peptide motifs that favor particular T lymphocyte polarization, such as Th2 to Th1, in a mouse model of leishmaniasis (68).

CTS also impact MHC class I-mediated antigen presentation. While MHC class I molecules usually present cytosolic peptide antigens, exogenous pathogen antigens can be presented by this complex *via* cross-presentation. Exogenous antigens captured by dendritic cells are initially processed in the endocytic pathway by CTS S followed by their final processing in the cytosol before being presented to CD8^+^ T lymphocytes (69).

In addition to T cell priming, CTS were found to regulate T lymphocyte polarization independent of APCs (70). The complement system integrates innate and adaptive responses and could influence the magnitude of T cell activation (70). Naive CD4^+^ T lymphocytes store C3 in endosomes that can be cleaved by CTS L generating C3a and C3b. The C3aR-mediated intracellular signaling induces low levels of mechanistic target of rapamycin (mTOR) activation that regulate T cell survival (70). During infection, downstream signaling pathways of mTOR facilitates Th1 cell polarization from naive T cells (70, 71).

## Cathepsins in the cytosol and in the nucleus

### Programmed cell death

CTS, as stated before, may be released into the cytosol by controlled or uncontrolled lysosomal membrane permeabilization (LMP), leading to lysosomal dependent cell death (reviewed in (16, 21)). Extensive permeabilization leads to necrosis (72) while a less drastic release induces apoptosis (16, 73–77). Stringent controlled release of CTS will allow the cells to survive and physiologic responses to CTS either in the cytosol or in the nucleus (16, 78–80). In the case of Salmonella infection a control of necrotic cell death was found associated with accumulation of active cathepsins in the nucleus (20). The additional control of cathepsin activity in these compartments depends on the balance and expression of natural inhibitors (78–80). For instance, it was demonstrated that the cytosolic inhibitor Spi2A protected memory CD8^+^ T lymphocytes from lysosomal breakdown and cell death by inhibiting CTS B activity (81). Spi2A is a serine protease inhibitor with an unusual role inhibiting cysteine cathepsins after lysosomal permeabilization (81). Consequently, this extends the lifespan of memory T cells.

The B-cell lymphoma-2 (Bcl-2) family proteins regulate the mitochondrial pathway of apoptosis. Interestingly, this family includes proteins with anti-apoptotic (e.g., Bcl-2 and Bcl-xL) and pro-apoptotic (e.g., Bax, Bak and Bid) activities and CTSs have direct roles in regulating several members of Bcl-2 proteins. For example, CTSs B, D, and L induce the activation of Bid, resulting in its translocation to mitochondria resulting in cytochrome C release and caspase activation. Moreover, CTS degrade anti-apoptotic proteins Bcl-2, Bcl-xL, Mcl-1, and XIAP (X-linked inhibitor of apoptosis), promoting apoptosis (82, 83).). In T lymphocytes, CTS D degrades Bax, triggering apoptosis *via* release of cystatin C and AIF (apoptosis-inducing factor) which directly activates caspase-8 (84–86). Finally, additional CTS (e.g. C, F, H, K, L, O, S, V, W, and X) also function as mediators of lysosomal cell death either in immune and non-immune cells ls (10).

Other forms of programmed cell death lead to inflammation through cell lysis as is the case of necroptosis and pyroptosis. Necroptosis requires the kinase activity of receptor-interacting serine/threonine kinase1 (Rip1), a protein that is cleaved by CTS B and S thus controlling inflammatory cell death (17). Pyroptosis is mediated by gasdermin, a pore forming protein dependent on inflammasome activation (87, 88). After LMP, CTS B and L are major inflammasome inducers that may lead to this form of cell death therefore enhancing the inflammatory responses (89).

### Cytosolic driven inflammation

As stated, CTS released to the cytosol following LMP are relevant activators of inflammasomes, structures involved in innate immune responses (18, 90). Among inflammasomes the NLRP3 inflammasome is a major complex of assembled proteins in response to LMP, DAMPS or PAMPS (91–94). It is required for caspase-1 activation in the cytosol that in turn cleaves pro-IL-1β to their inflammatory mature form (90). Although CTS B and L have been associated with NLRP3 inflammasome activation, several siRNA experiments implicated CTS S and X (Z), particularly in contexts were they may compensate the lake of activity of CTS B and L (18, 95).

Inflammation is concomitant with migration of immune cells into tissues, such as lymphocytes and macrophages. CTS X is highly expressed in immune cells namely macrophages, dendritic cells and T lymphocytes (79). Its function has been associated to inflammatory responses such as cell adhesion, cell migration and phagocytosis. Some of these processes are the result of CTS X activation of transmembrane surface proteins, β2 integrins.  (96–98). To do so CTS X cleaves the last four amino-acids contained in the cytosolic part of C-terminal region of β2 integrins, either Mac-1 receptor in macrophages and dendritic cells, or LFA-1 in T lymphocytes. Activation of Mac-1 enhances adhesion of macrophages and dendritic cells to extracellular matrix (ECM), improving phagocytosis and subsequent maturation of dendritic cells, a process essential for antigen processing and presentation (96). Activation of LFA-1 causes proliferation and tissue homing of T lymphocytes characteristic of acute and chronic inflammations (98).

### Regulation of transcription

CTS traffic to the nucleus has been associated to activation of transcription factors that control cell proliferation and differentiation (19, 22). Among transcription factors, CDP/Cux/Cut is activated by CTS L enabling accelerated cell cycle progression and carcinogenesis (19). Nuclear activity of CTS L was associated to an abnormal nuclear trafficking of the full length protein when stefinB, a CTS L inhibitor, is absent (19).

CTSs K and S were shown to interfere with nuclear membrane transport and control TGF-β signaling, leading to ECM synthesis required for cell growth and tissue fibrosis that often occurs during infections (99). They modulate the nuclear import of Smad proteins transcription factors that in turn regulate the expression of profibrotic genes such as collagen and fibronectin. In opposition, CTS B and L in nuclear membrane inhibit the effects of CTS K and S leading to decreased TGF-β signaling (99). This fibrotic pathological response may indeed be mitigated by extracellular CTS while promoting ECM degradation and helping tissue repair (100).

## Cathepsins in the extracellular space

CTS emerge therefore as relevant players in the extracellular space as full degrading enzymes of ECM components, but the paradigm is now changing to enzymes that can specifically modify other extracellular proteins. Their secretion and activity are often dysregulated during inflammatory responses including infection [recently reviewed in (28)].

### ECM remodeling

The structure of the ECM is dynamic and depends on the equilibrium between synthesis and degradation of a multitude of proteins (collagens, fibronectin, elastins), growth factors, proteoglycans, among others (11). ECM is vital to cell support and tissue integrity and has a series of regulatory functions. CTSs K, S, and V possess strong collagenolytic and elastolytic activities suggesting their involvement in ECM remodeling (2, 11). The best studied is CTS K that degrades type I collagens being essential for normal bone resorption (101). Other targets of CTS are cell adhesion contacts, influencing epithelial barriers, and cell adhesion to ECM, leading to changes in cell growth, cell migration, angiogenesis (102) and tissue repair (11, 103, 104). CTSs B and L have been shown to be released by lysosomal exocytosis playing a role in repair of the plasma membrane (105). CTS B, released from keratinocytes, attaches to cell surface where it is known to be involved in keratinocyte migration by degrading components of ECM during wound healing (106).

### Extracellular driven inflammation during infection

CTS secretion to ECM is usually high during infection. Microorganisms are sensed by innate immune receptors in mucosal cells that respond with an increased secretion of a myriad of proteases including antimicrobial peptides and CTS all having antimicrobial effects (28, 104, 107). This is the case of CTS K, highly expressed in intestinal Goblet cells, or CTS G, secreted from Paneth cells, that contributes to pathogen and microbiota control, and epithelial barrier repair (104, 107). In bronchial mucosa a protective effect was attributed to CTSs B and L (28) while CTS S, expressed mainly in macrophages, may favor the motility of cilia by preventing unspecific binding with airway circulating proteins (6).

However, the proteolytic activity of CTS may also aid infections by cleaving viral envelope proteins activating their receptor-binding or fusogenic activities, thus favoring viral infection (108–111). In chronic inflammatory conditions high concentrations of CTSs B, L, and S have been shown to cleave and inactivate several proteases, impairing their antimicrobial properties (2, 112, 113). The extensive destruction of lung parenchyma in tuberculosis is related with high levels of CTSs K, S, and V (114). CTSs G and D favor autolysis inside tuberculosis granulomas contributing to their liquefaction and disruption thus facilitating pathogen dissemination (28, 115).

The ECM breakdown products produced by extracellular proteases, including cathepsins, may act themselves as DAMPS, leading to the activation of NLRP3 inflammasomes exacerbating tissue inflammatory responses (116).

Extracellular CTS are also able to process cytokines and chemokines. CTSs L, S, and K, were shown to activate the glutamate-leucin-arginine motif (ELR) CXC ELR and inactivate non-ELR (CXCL9–12) chemokines thereby contributing to leukocyte recruitment during protective or pathological inflammation (117). CTS L secreted from fibroblasts and CTS G secreted from macrophages, neutrophils, and epithelial cells are activators of IL-8 (CXCL8). IL-8 acts both as a strong neutrophil chemoattractant, and as a proinflammatory cytokine (118, 119). In addition, CTS G activates IL-1β and TNFα as well as various signaling receptors (120). In contrast, CTS G can reduce dramatically the activity of IL-6 in fluids from inflammatory sites (121).

In the extracellular space CTS are able to cleave ectodomains of receptors and cell adhesion molecules at the cell surface, influencing by this mechanism several signaling pathways (122, 123). CTSs L and S secreted from macrophages were shown to shed CAM adhesion proteins and receptor tyrosine kinases (123). Dysbiosis-induced disruption of the epithelial barrier was found to be related with ectodomain activation of protease-activated receptor 4 (PAR 4) by neutrophil CTS G (124).

## Discussion

Cathepsins spatial localization is associated with distinct key roles of immune responses, with strong implications for infection control and inflammation resolution. Thus, CTS manipulations within these spatial contexts constitute potential targets for the development of new therapeutic strategies to fight infections, particular for those pathogens that developed drug resistance mechanisms to conventional treatments. The enhancement of their activity in situations where pathogen survival relies on their inhibition (e.g., drugs targeting autophagy) may help pathogen eradication from infected cells. Conversely, when infection results in poor antigen presentation, manipulation of CTS activity may improve the adaptive response and vaccine efficacy. Pathological inflammation is often a consequence of an infection. Targeting the control of inflammatory pathways may help to prevent or resolve tissue destruction and fibrotic events. There is still plenty to be investigated in this very promising area of research to fight the increasing threat of infections.

## Author contributions

Conceptualization and writing: EA. Review and editing: DP, JA-P, and MM. Image: EA. Supervision and funding acquisition: EA. All authors contributed to the article and approved the submitted version.

## Funding

This study was supported by grants from the National Foundation for Science, FCT Fundação para a Ciência e Tecnologia – Portugal, PTDC/SAU-INF/28182/2017 to EA, UIDB/04138/2020 (to IMed-ULisboa).

## Acknowledgments

ADEIM-FFUL (Associação para o Desenvolvimento do Ensino e Investigação em Microbiologia).

## Conflict of interest

The authors declare that the research was conducted in the absence of any commercial or financial relationships that could be construed as a potential conflict of interest.

## Publisher’s note

All claims expressed in this article are solely those of the authors and do not necessarily represent those of their affiliated organizations, or those of the publisher, the editors and the reviewers. Any product that may be evaluated in this article, or claim that may be made by its manufacturer, is not guaranteed or endorsed by the publisher.
